# Availability, access, analysis and dissemination of small-area data

**DOI:** 10.1093/ije/dyz051

**Published:** 2020-04-15

**Authors:** Susan Hodgson, Daniela Fecht, John Gulliver, Hima Iyathooray Daby, Frédéric B Piel, Fuyuen Yip, Heather Strosnider, Anna Hansell, Paul Elliott

**Affiliations:** 1 MRC-PHE Centre for Environment and Health, School of Public Health, Imperial College London, London, UK; 2 UK Small Area Health Statistics Unit, MRC-PHE Centre for Environment and Health, Imperial College London, London, UK; 3 Environmental Health Tracking Section, National Center for Environmental Health, Centers for Disease Control and Prevention, Atlanta, USA

**Keywords:** Small-area studies, open data, remote sensing, environment and health

## Abstract

In this era of ‘big data’, there is growing recognition of the value of environmental, health, social and demographic data for research. Open government data initiatives are growing in number and in terms of content. Remote sensing data are finding widespread use in environmental research, including in low- and middle-income settings. While our ability to study environment and health associations across countries and continents grows, data protection rules and greater patient control over the use of their data present new challenges to using health data in research. Innovative tools that circumvent the need for the physical sharing of data by supporting non-disclosive sharing of information, or that permit spatial analysis without researchers needing access to underlying patient data can be used to support analyses while protecting data confidentiality. User-friendly visualizations, allowing small-area data to be seen and understood by non-expert audiences, are revolutionizing public and researcher interactions with data. The UK Small Area Health Statistics Unit’s Environment and Health Atlas for England and Wales, and the US National Environmental Public Health Tracking Network offer good examples. Open data facilitates user-generated outputs, and ‘mash-ups’, and user-generated inputs from social media, mobile devices and wearable tech are new data streams that will find utility in future studies, and bring novel dimensions with respect to ethical use of small-area data.

**Key Messages**

- Availability of spatially resolved data is increasing, with new data being put to use in small-area environment and health studies.
- Access to data is supported by open data initiatives, however the tightening of information governance and greater autonomy over use of personal data will impact health research.
- Analysis tools that support and audit the ethical and legal use of health data are likely to find increasing utility in small-area and multi-cohort studies.
- Dissemination of data to a wide audience can support public understanding of environment and health research.
- User-generated data, from social media, smart phones and wearable tech, will support future environment and health studies.

## Introduction

This paper reflects on some of the issues associated with the availability, access, analysis and dissemination of small-area data. Small-area data in this context refers to data describing characteristics—e.g. health, environment, demographic, economic—of a defined geographic area. The definition of ‘small area’ will vary, study by study, depending on access and availability of data (more below), but also the scale appropriate to the analyses to be undertaken (which is contingent on rarity of outcome and/or the size of the population under study).^1^ Ideally the small areas will describe relatively homogeneous populations in terms of exposures and/or key variables of interest, as it is this characteristic of a small-area study that can reduce components of ecological bias.^2^ The small-area approach, then, benefits from the efficiencies of being able to utilize data collected and made available at a range of postal, census and/or administrative geographies, while minimizing the bias inherent in the ecological approach. Here, we offer our view on upcoming opportunities and challenges in this field, drawing on our 30 years of experience working at, or with, the UK Small Area Health Statistics Unit (SAHSU).

## Availability of small-area data

Spatially resolved datasets are becoming increasingly available for research, although it is important that the quality of the data, and their limitations, are well understood in order to be able to make meaningful inferences from their use.^3^^,^^4^ In the UK, existing public data are becoming more readily available via platforms such as the UK Open Data portal (data.gov.uk), which currently provides free access to >40 000 datasets from more than 1000 sources, under the principle that all information created by the central government, local authorities and public sector bodies should be made available for re-use. Similar web portals hosting open government data are available, for example, in the USA (www.data.gov), France (www.data.gouv.fr) and Singapore (www.data.gov.sg).^5^

**Figure 1.**
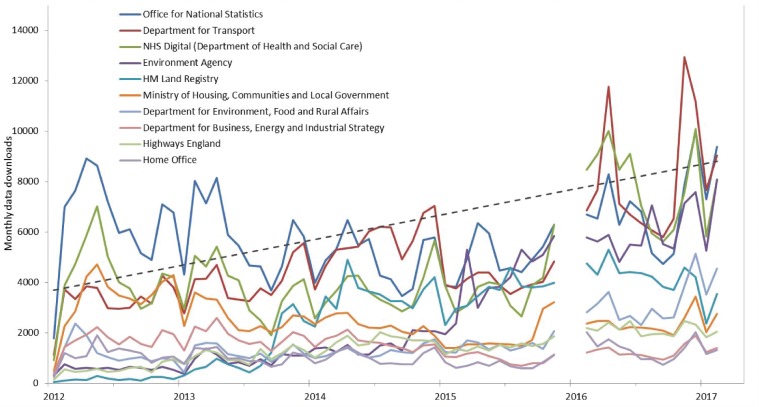
Usage statistics (downloads), by publisher for each month (December 2012–March 2018) from Data.gov.uk for top 10 data publishers, with linear trend line for total monthly downloads. Data generated April 5, 2018, from Google Analytics. Contains public sector information licensed under the Open Government Licence v3.0.

Such data are being accessed and downloaded with increasing frequency for a wide range of purposes. Figure 1 shows monthly data downloads for the top 10 publishers from the Data.gov.uk portal. There is also evidence emerging to suggest an increasing use of open government data specifically for scientific research, most prominently in high-income countries. Amongst 1229 studies using open government data investigated in a review by Yan and Weber,^6^ 25.5% and 11.6% used open access data from the UK and USA, respectively; however, 7% and 6.3% of studies used data from India and Kenya, suggesting open data are also a valuable scientific resource in some low- and middle-income countries.

### Health data

As seen in Figure 1, open data from the Office for National Statistics and NHS Digital are frequently downloaded from the Data.gov.uk portal. NHS digital publish more than a thousand datasets on health and social care provided by the National Health Service (NHS). These datasets describe aspects of primary care, secondary care, emergency care, community services, maternity services, mental health, social care, clinical audits and disease registers, prescribing, population health, the NHS workforce and estates, clinical indicators, healthcare resources, data standards and data quality.^7^ The Office for National Statistics (ONS) provides annual statistics on births and deaths in England and Wales, with near complete ascertainment.^8^^,^^9^ The ONS also holds national data on child health, disability, drug use, alcohol and smoking, and life expectancy. Public Health England are custodians of the National Cancer Registration and Analysis Service, which provides data on 30 years of cancer registrations.^10^

In addition to these established datasets that are well used in research, new health datasets are being made available for research. For example, general-practice-level prescribing data, detailing all medicines, dressings and appliances prescribed and dispensed each month in England since August 2010 (https://openprescribing.net/), have found utility in studies assessing health service inequalities^11^ and in work linking environmental exposures to volume and cost of prescribing.^12^ Hospital outpatient data for England was accredited as a national statistic in 2008. This valuable resource, detailing 119 million outpatient appointments in the year February 2017 to January 2018^13^ is now also being used in research alongside the more established inpatient data.^14–16^ The quality of these data, in terms of ascertainment, has however been found lacking (e.g. self-harm episodes were under-ascertained when compared with local bespoke data collection methods^16^) and the diagnosis fields were reported to be too infrequently populated to be useful (although it was possible to use date and specialty of appointment to study hospital-service use following heart failure^15^).

### Environmental data

With respect to environmental data describing the distribution of levels of pollutants, the increased availability of highly spatially resolved data, for large geographic areas, has supported the development of harmonized exposure assessment on a national/international scale, for certain pollutants. This up-scaling and harmonization supports cross-country comparisons and increases the statistical power to look at subtle effects and/or rare outcomes. Recent air pollution exposure assessments on national and international scales have been based on land use regression (LUR) modelling approaches that combine two types of data: (i) a chemical transport model or information from satellite data [e.g. aerosol optical depth (AOD)] to describe regional/background concentrations of particulate pollution, typically with a granularity of 1–10 km, and (ii) localized spatial predictors summarized in circular buffers of varying radii from tens to hundreds of metres (e.g. lengths of roads, traffic intensity, area of housing, industry, green space etc.) or distance to source. A spatially distributed network of air pollution monitors is required to develop and validate the LUR model. Using this approach, de Hoogh *et al*.^17^ (2016) developed air pollution surfaces at a resolution of 100 meters for the whole of western Europe using data from the European network of air pollution monitoring sites (Airbase), data from a chemical transport model (NO_2_) and AOD [particulate matter with an aerodynamic diameter <2.5 micrometers (PM_2.5_)] for background sources, pan-European land cover (CORINE), and national models of road traffic. This LUR model explained 60% of the variability in PM_2.5_ concentrations (48% related to AOD; 12% related to local predictors); without information on AOD, the local spatial predictors only explained 38% of the variability in PM_2.5_ concentrations. Such air pollution surfaces produced on a 100 m grid provide sufficient granularity to characterize the variability in exposures within and between small areas. Similar examples include NO_2_ for Australia,^18^ the USA,^19^ and a global model of NO_2_ albeit with a reduced spatial resolution in some countries.^20^

Whereas such models have been used for estimation of outdoor pollutant concentrations at specific locations, e.g. residential address, for defined periods, such as annual^21^^,^^22^ or trimester-specific estimates for birth outcomes,^23^^,^^24^ they are not suitable for estimating time-varying exposures at a much higher temporal resolution, e.g. with respect to daily commuting patterns, nor for indoor exposures. Methods to address these areas require further development, as discussed further below.

Similar advances due to better accessibility to environmental data have been observed in other fields as well. Noise is largely a local pollutant, propagating over tens to hundreds of metres in most cases. Accurate noise modelling requires detailed geographical data to characterize source emissions (e.g. individual road links with time-varying traffic flows/speeds) and noise propagation (e.g. geometry and height of individual buildings, and highly resolved land cover data differentiating between different types of man-made and natural surfaces) and is computationally demanding. These considerations have presented a substantial challenge for modelling noise over large geographical areas, especially for national- or international-scale epidemiological studies. Although proprietary software (e.g. CadnaA, SoundPlan) has long been available, open-source software has only recently been developed,^25^^,^^26^ with some simplifications both to data requirements (e.g. using average building height and fewer categories of land cover) and emissions/propagation models to serve the needs of large-scale studies. Noise levels are normally calculated at one or more façade locations of individual dwellings,^26–28^ except, for example, in EU strategic noise mapping in agglomerations (>100 000 residents) where surfaces are produced. Morley *et al*.^25^ describe the implementation of a version of the CNOSSOS-EU (Common NOise aSSessment MethOdS) noise modelling framework, which was used to undertake harmonized noise exposure assessment for four large cohort studies (UK Biobank and EPIC Oxford in the UK, Lifelines in the Netherlands and HUNT in Norway) participating in the EU FP7 funded Biobank Standardisation and Harmonisation for Research Excellence in the European Union (BioSHaRE-EU) project. This work found road traffic noise exposure to be associated with blood biochemistry^29^ and heart rate,^30^ but not with incident cardiovascular disease,^31^ blood pressure^30^ or asthma prevalence.^32^

The examples above highlight the benefits of increased data availability and processing power to generate improved resolution exposure models with greater geographical coverage. However, the resultant exposure metrics still represent outdoor exposure concentrations that may not reflect personal exposure. The benefits of studying a larger population, i.e. the ability to study rare diseases, undertake sub-group analyses and assess interactions, need to be balanced by the loss of power/interpretability introduced when using such proxy measures of personal exposure. As discussed later, geo-location data from mobile devices and sensor data from wearable tech might permit modelled outdoor exposures to be calibrated to individuals and/or sub-groups of the population to better reflect personal exposures.

Use of satellite-derived data is of particular importance for low- and middle-income countries (LMICs) where routinely collected environmental data might not be available, as well as for global studies such as those conducted by the Global Burden of Disease project (GBD, www.healthdata.org/gbd ).^33^ Remotely sensed data are now extensively used for epidemiological purposes to evaluate global risks to human health via climate change or environmental pollution.^33^ They are also crucial in identifying suitable habitats for vector-borne diseases and thereby to monitor, control and prevent infectious diseases such as malaria and neglected tropical diseases.^34–36^ The Normalized Difference Vegetation Index (NDVI), for example, is a widely applied indicator from remotely sensed data to evaluate the extent of live green vegetation. This has been used to track agricultural patterns to estimate the burden of child malnutrition in African countries^37^ and, more often in developed countries, to estimate exposure to salutogenic urban green space (e.g.).^38^ Satellite-derived environmental data have also contributed to a better understanding of distributions and geographical patterns of infections and chronic diseases that are largely driven by environmental factors such as temperature, soil characteristics and land use. Examples include soil-transmitted helminth infections in South America, where remotely sensed data have allowed spatial distributions of infection prevalence to be predicted, to support the targeting of populations for treatment,^39^ and Zika virus, where remotely sensed data contributed to vector distribution modelling, suggesting that 2.17 billion people inhabit areas that are environmentally suitable for Zika transmission.^40^

### Socio-economic data

Deprivation is strongly associated with risk of disease and with disease risk factors, with inequalities in socio-economic status accounting for half of the inequalities in some diseases.^41^ As such, it is important to gather area-level information on deprivation so that adjustment for this key confounding variable can be made. Within the UK, several area-level measures of deprivation, derived from census data, are available. The most commonly used include (i) the Carstairs Index, which comprises four indicators of material disadvantage—lack of car ownership, low occupational social class, overcrowded households and male unemployment^42^; (ii) Townsend Deprivation Score, which also includes four indicators of deprivation—unemployment, non-car ownership, non-home ownership and overcrowding,^43^ and (iii) the Index of Multiple Deprivation (IMD), which combines information from seven domains of deprivation—income, employment, education/skills/training, health and disability, crime, housing and services, and living environment.^44^ The choice of deprivation measure will depend on the geographic scale and/or time frame of analysis (not all measures are able to be calculated for all geographic areas, nor from each census), but also on measures used in previous studies (if direct comparison is important), and on the composition of the index [e.g. if it is important to adjust (or not) for health (which is a component of IMD)]. Whichever measure is used, it should be remembered that these are all ecological measures of deprivation, i.e. describe area-based, not individual circumstance.

### Population data

Prerequisite for the accurate identification of populations at risk from geographically varying diseases is detailed, high spatial resolution information on human population distributions. In developed countries, various methods have been put forward to obtain accurate information on population at the small-area scale.^45^ But this is a particular challenge in LMICs where extensive mapping resources and detailed population counts are often lacking. Recent developments such as the WorldPop project (http://www.worldpop.org.uk/) have tried to fill this gap using geospatial data from satellites on land cover and light-at-night, together with locally available census information, to estimate small-area population distributions globally.^46^

The health, environment and socio-economic data holdings at SAHSU, to support small-area health analyses within the UK, are summarized in Table 1.

**Table 1.** Health, environment and socio-economic data holdings of the Small Area Health Statistics Unit (SAHSU) database (more details at: https://www.sahsu.org/content/sahsu-database)

Dataset	Geographic extent	Provider
Health data		
Hospital Episodes Statistics (HES) Admitted Patient Care	England	NHS Digital
HES Accident and Emergency	England	NHS Digital
HES Critical Care	England	NHS Digital
Cancer registrations	England	Office for National Statistics
Cancer registrations	Wales	The Welsh Cancer Intelligence and Surveillance Unit
Deaths registrations	England and Wales	Office for National Statistics
Birth and still births registrations	England and Wales	Office for National Statistics
Local Congenital Anomaly Registers	Regions of the United Kingdom and the Republic of Ireland	British Isles Network of Congenital Anomaly Registers
Scottish births, mortality and congenital anomalies	Scotland	NHS Scotland (Information Services Division)
Environment data		
Land Cover Map	Great Britain	Centre for Ecology and Hydrology
CORINE Land Cover	EU	European Environment Agency
Agricultural Census	England	Edinburgh
Air temperature	England and Wales	SAHSU/British Atmospheric Data Centre
Sunshine duration	England and Wales	SAHSU/British Atmospheric Data Centre
Light emissions	EU	SAHSU/NOAA National Geophysical Data Centre
NO_2_ and PM_10_	Great Britain	RGI
NO_2_ and PM_10_ background concentrations	EU	APMOSPHERE EU project
NO_2_ and PM_10_	EU	ESCAPE EU project
Historic black smoke and SO_2_	Great Britain	CHESS Wellcome project
NO_2_	Great Britain	CHESS Wellcome project
Heavy metals (lead and cadmium) in soil	England and Wales	SAHSU/Countryside Survey
Road Traffic Noise	Great Britain	BioSHaRE EU project
Road Traffic Noise	London	TRAFFIC NERC project
Socio-economic data		
Carstairs Index	Great Britain	Office for National Statistics (Census data)
Townsend Index	England	Office for National Statistics (Census data)
Index of Deprivation	England, Wales, Scotland, Northern Ireland	Ministry of Housing, Communities & Local Government
Urban–rural classifications	Great Britain	Office for National Statistics

CHESS, Chronic Health Effects on Smoke and Sulphur; ESCAPE, European Study of Cohorts for Air Pollution Effects; HES, Hospital Episodes Statistics; NERC, Natural Environment Research Council; NOAA, national oceanic and atmospheric administration; RVI, Ruimte voor Geo-Informatie.

## Access to small-area data

Against this backdrop of increased availability of health and environmental data, there has been a tightening of information governance regulation, particularly in the UK and EU. For example, in January 2014, the UK government formalized the right of patients in England to request that their confidential information is not used beyond their own care and treatment. Patients who ‘opt-out’ will no longer have their personal confidential information appear in data disseminations from data providers; their information can only be made available in anonymized form (so that the individuals are not identified in the data). Data from patients who opt-out may not therefore be represented in epidemiological or health service research studies where personal confidential data are required for data linkages. Patients choosing to opt out are demographically and geographically clustered, and increasing in number over time. For instance, opt-out rates were reported to be significantly higher in older versus younger patients, in female versus male patients, and differed by ethnicity and area-level deprivation.^47^ As age, sex, ethnicity and socio-economic status are important confounders in health studies, differential loss of numerator and/or denominator data due to opt-outs is likely to bias observed associations between risk factors and health outcomes.

Beyond the UK, Australia is currently debating the pros and cons of opting in/out of the electronic ‘My Health Record’, that advocates claim will benefit patients via the sharing of key health information, and lead to efficiencies in healthcare, while others are concerned about data security issues.^48^ In 1995 Denmark established a system whereby citizens could opt-out of having their details shared for research projects, however this option was revoked in 2014 when an estimated 16% of the population had opted out.^49^ Information on how data are used, who has access to sensitive information, and the safeguards in place need to be carefully communicated if the public are to be convinced of the case for sharing health data for research. The growing demand for patients to control their data in the era of big data is understandable, but there are important consequences for public health research that relies on the availability of comprehensive datasets and patient identifiable data to improve health and social care.

The EU General Data Protection Regulation (GDPR), enforced May 25, 2018, aims to harmonize data privacy laws across Europe (replacing the Data Protection Act 1998 in the UK), with important implications for health research. Health data fall within the ‘special category’ of personal data, and require, in addition to a ‘lawful basis’ for processing, that further conditions are met before use. Of relevance here, data processing for preventative or occupational medicine, the management of health or social care systems and services, and public health are included as further conditions for processing such special category data. The harmonization of data protection regulation might improve and facilitate data sharing across the EU, supporting multi-country studies.^50^

Although data sharing between countries within the EU might be supported by the GDPR, the ethical sharing of data between different jurisdictions presents a challenge to the scaling-up of research. ‘Compute to data’ methods provide one approach to avoid the need for physical sharing of data. As an example, DataSHIELD (www.datashield.ac.uk/) has been developed to support the non-disclosive sharing of information. This approach can facilitate research in circumstances where data governance might prevent the release of data and/or the combination of multiple datasets for unified analysis, or where data providers are happy to share information but do not wish to cede control of the governance of those data and/or the intellectual property they represent by physically sharing.^51^ Such an approach was used in the BioSHaRE-EU project to permit the combined individual-level analysis of harmonized data from participants from several European cohorts, some of which were held by cohort custodians and which were queried remotely. For example, this approach was used to assess the associations between ambient air pollution and traffic noise and adult asthma prevalence (using data from 646 731 participants from HUNT3, Lifelines and UK Biobank^32^) and cardiovascular risk factors (in 144 082 participants from HUNT3 and Lifelines^29^) and air quality on wheeze/shortness of breath (in 377 954/173 560 participants from Lifelines and UK Biobank^52^).

## Analysis of small-area data

The need for user-friendly tools, capable of processing a large amount of information and supporting the linkage of datasets, their analysis and visualization is also increasing. One such tool is the SAHSU ‘Rapid Inquiry Facility’ (RIF), developed in the late 1990s^53^ and refined for use in the EU.^54^ The RIF software was then adapted and enhanced for use in the US Centers for Disease Control and Prevention (CDC) National Environmental Public Health Tracking Network, as one of several tools used by the Tracking Program.^55^ The RIF facilitates environmental health analysis, by linking health, environmental, socio-economic, population and geographic data. The RIF supports disease mapping studies (standardized disease rates and relative risks across a user-specified area to explore the spatial distribution of disease) and risk analysis (to investigate whether a putative exposure source is associated with adverse health outcomes in the exposed population). The latest version of the RIF (RIF 4.0) is an open source, freely accessible web platform on a spatially enabled database, PostGIS.^56^ In addition to the integration of advanced methods in statistics, exposure assessment and data visualization, the RIF also generates an audit trail, to facilitate adherence to data protection and information governance requirements mentioned above.

The RIF has been used to assess, for example, kidney disease mortality following a historic industrial contamination incident in the UK,^57^ mortality from cardiovascular and cerebrovascular disease and drinking water hardness in Spain,^58^ the association between deprivation and circulatory system disease mortality in Hungary^59^ to investigate cancer rates in residents living over contaminated groundwater plumes near an Air Force Base in Utah, USA,^60^ and to explore the geographic variation of cancer incidence at the neighbourhood level in Ontario, Canada.^61^

Tools, such as the RIF, can facilitate small-area analysis, but cannot replace the need for epidemiological, geographical and statistical input in the planning, analysis and interpretation of small-area analyses. This expertise is necessary to ensure that: (i) appropriate health, exposure, covariate and population data are selected; (ii) associations are assessed at a meaningful geographic (and temporal) scale; (iii) an appropriate statistical approach is applied; and (iv) the resulting output is interpreted, with full appreciation of any data quality issues and an understanding of the limits of the small-area approach. Considerations regarding data choice, study area, time period and analytic method are further discussed by Piel *et al,*^1^ and a review of the main methodologic issues associated with the small-area approach is presented in Beale *et al*.^2^

## Dissemination of small-area data

Development of user-friendly interfaces and visualizations has allowed small-area data to be disseminated to a wide audience including researchers, health professionals, policy makers and the public. The Environment and Health Atlas for England and Wales and US CDC National Environmental Public Health Tracking Network website, are two examples where health and environment data are presented, along with supporting information, to facilitate public understanding.

**Figure 2.**
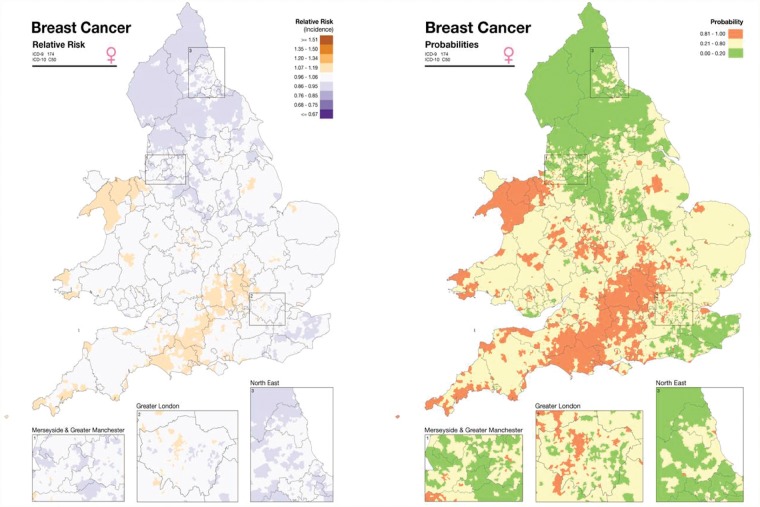
Disease map from the Small Area Health Statistics Unit (SAHSU) Environment and Health Atlas for England and Wales (www.envhealthatlas.co.uk). Left-hand side: smoothed relative risk of female breast cancer incidence in England and Wales, adjusted for age and deprivation, 1985–2009; right-hand side: posterior probabilities for female breast cancer incidence in England and Wales, adjusted for age and deprivation, 1985–2009. Contains National Statistics and Ordnance Survey data © Crown copyright and database right 2013. Cancer incidence for Wales was supplied by welsh cancer intelligence and surveillance unit (WCISU).

The SAHSU’s Environment and Health Atlas for England and Wales (www.envhealthatlas.co.uk) provides interactive maps for a range of health conditions and environmental agents at the small-area scale (census ward level, average population 6000) in England and Wales. The maps were developed as a resource for the public, researchers and those working in public health and policy, to support the understanding of the geographic distribution of environmental agents and health conditions in England and Wales. To facilitate interpretation, the Atlas presents age- and deprivation-adjusted disease risks for males and females separately, with statistical smoothing to adjust for chance fluctuations in disease risk that can occur when using small numbers of cases or small populations (Figure 2). The print version of the Atlas includes additional interpretative text, and a detailed explanation of the statistical methods used.^62^ To ensure that the Atlas was useful for the target audience, SAHSU worked closely with the independent charity, Sense about Science (senseaboutscience.org/), who brought together a range of specialists including epidemiologists, health geographers, statisticians, medical doctors, journalists, science communicators, representatives from government organizations, local government as well as interested members of the public. They critically assessed the Atlas material and highlighted issues concerning the display of maps and clarity of content, leading to improvements in presentation and interpretability.

The mission of the US CDC National Environmental Public Health Tracking Program is to provide information from a nationwide network of integrated health and environmental hazard and exposure data to drive actions to improve the health of communities.^63^^,^^64^ In collaboration with partners, the Tracking Program identifies priority environmental health issues, determines key surveillance questions, and evaluates the utility of existing data for answering the question and informing the issue. Selected data are integrated into the National Environmental Public Health Tracking Network and used to (i) describe temporal and spatial trends in disease and potential environmental exposures, (ii) identify populations most affected, (iii) generate hypotheses about associations between health and environmental exposures, and (iv) develop, guide, and assess the environmental public health policies and interventions aimed at reducing health outcomes associated with environmental factors. Gaps in data are addressed by developing standards for new data collections, models, or new methodologies for using existing data, or by expanding the utility of non-traditional public health data. The Tracking Network (https://ephtracking.cdc.gov) permits viewers to explore interactive maps, tables and charts, view information by location (county), and visit state and local tracking websites. The Tracking Program is currently working to improve the spatial resolution (to geographic units smaller than county) of the publicly disseminated data to better address local-level issues. Efforts must balance the need for small-area health data with the need to protect confidentially and produce stable, reliable disease rates. Additionally, the Tracking Program is enhancing the Tracking Network to facilitate the delivery of real-time data to mitigate acute exposures to elevated levels of environmental hazards.

User-generated output, e.g. mash-ups and web applications that combine data from multiple sources and provide additional information and/or functionality, are also supporting the dissemination of small-area data. For instance, the Environmental Research Group of King's College London have developed the London Air app (www.londonair.org.uk), which displays up to date air pollution levels based on measurements taken within the previous hour from monitoring stations that comprise the London Air Quality Network, combined with a detailed model, to show a prediction of air quality at a 20 m resolution across the whole of Greater London.

## Future opportunities and challenges

User-generated inputs, including data from web searches and social media (e.g. for influenza surveillance^65^^,^^66^), accelerometer data and geolocations from mobile devices (e.g. for assessing active transport^67^) and physiological and environmental sensor data from wearable tech (e.g. for assessing air pollution exposure^68^) are opening up new data streams for future studies. Most of these datasets are currently underused for epidemiological studies, but combining, for example, time-activity data from travel surveys, Global Positioning Systems (GPS) devices and/or accelerometer data with high-resolution modelled exposure surfaces will permit improved characterization of exposure to spatially and temporally varying risk factors. For example, travel survey data were used in conjunction with estimated micro-environmental concentrations of PM_2.5_, black carbon, and NO_2_ to characterize air pollution exposures for 89 000 individuals in Hong Kong, with ‘dynamic’ exposure estimates differing significantly from the ‘static’ exposure assigned to residential address.^69^ Smaller studies have also trialled the use of smart phone based GPS/physical activity data, along with highly spatially and temporally resolved air pollution mapping, e.g. to better understand activities contributing to air pollution exposure in Barcelona.^70^ The availability of, for example, accelerometer-measured physical activity in >100 000 participants of the UK Biobank study^71^ and >27 000 children in the International Children’s Accelerometry Database^72^ indicates that these data can be collected at scale.

New data brings new challenges, including demographic bias due to access/availability/trust in digital tools and apps,^73^ along with considerations in establishing terms for ethical data use, that will need to be carefully considered.

## Funding

The work of the UK Small Area Health Statistics Unit is funded by Public Health England as part of the MRC-PHE Centre for Environment and Health, funded also by the UK Medical Research Council.

The findings ad conclusions in this article are those of the authors and do not necessarily reflect the views of Public Health England or the Department of Health, nor represent the official position of the Centers for Disease Control and Prevention.

**Conflict of interest:** None declared.
